# Evaluating the Potential of Multi-Walled Carbon Nanotube-Modified Clay as a Landfill Liner Material

**DOI:** 10.3390/ma16247705

**Published:** 2023-12-18

**Authors:** Xibin Liu, Zhengfa Chen, Lin Qi

**Affiliations:** 1 School of Environmental Science and Engineering, Changzhou University, Changzhou 213164, China; liuxibinczu@126.com (X.L.); qilincczu@126.com (L.Q.); 2 School of Urban Construction, Changzhou University, Changzhou 213164, China

**Keywords:** multi-walled carbon nanotubes, clay, geotechnical properties, adsorption properties, microstructure

## Abstract

In this paper, the feasibility of multi-walled carbon nanotube (MWCNT)-modified clay as a landfill liner material is investigated. Experiments were conducted on the modified clay with 0.5%, 1%, and 2% MWCNTs. The effects of the MWCNTs on the compaction characteristics, permeability coefficient, stress–strain curve, peak deviation stress, shear strength parameters (internal friction angle and cohesion), microstructures, and adsorption performance of the clay were analyzed. The results showed that the optimum moisture content (OMC) increased from 16.15% to 18.89%, and the maximum dry density (MDD) decreased from 1.79 g/cm^3^ to 1.72 g/cm^3^ with the increase in MWCNTs. The permeability coefficients firstly fell and then gradually rose as the MWCNTs increased; the minimum permeability coefficient was 8.62 × 10^−9^ cm/s. The MWCNTs can also effectively increase the peak deviation stress of the clay, and at the maximum level, the peak deviation stress was increased by 286%. SEM images were processed using the Pore and Crack Analysis System (PCAS), and the results showed that the appropriate amount of MWCNTs could fill the pores and strengthen the clay structure. The effect of the MWCNT-modified clay on the adsorption performance of common heavy metal ions Cd^2+^, Mn^2+^ and Cu^2+^ in landfill leachate was analyzed by batch adsorption tests. The maximum adsorption capacities (*Q*_max_) of Cu^2+^, Cd^2+^ and Mn^2+^ in the 2% MWCNT-modified clay were, respectively, 41.67 mg/g, 18.69 mg/g, and 4.97 mg/g. Compared with the clay samples without MWCNTs, the adsorption properties of Cu^2+^, Cd^2+^, and Mn^2+^ were increased by 228%, 124%, and 202%, respectively. Overall, the results suggest that MWCNT-modified clays have the potential to be suitable barrier materials for the construction of landfills.

## 1. Introduction

The best way to dispose of municipal solid waste (MSW) is through landfills because of their low construction and operating costs, along with good hygiene [1]. These domestic wastes typically consist of numerous varieties of heavy metals, and these heavy metal ions can seep into the groundwater and beneath-ground soil with the leachate generated from the landfill, causing damage to human health and the environment [2,3]. To minimize the contamination of groundwater and soil by landfill leachate, liner systems are commonly used as a key component of landfill hydraulic and chemical barriers [4].

On the basis of previous studies [5,6,7], the following three points are usually taken into consideration when selecting liner materials: (I) a low permeability coefficient (<1 × 10^−7^ cm/s) to reduce the movement of leachate to the groundwater and beneath-ground soil, (II) sufficient compressive strength (>200 kPa) to withstand the additional pressure exerted by MSW, and (III) retain as much of the harmful pollutants as possible in the leachate. Compacted clay is frequently utilized as a landfill liner material because of its low permeability, high strength, and adsorption of contaminants [8,9]. Nonetheless, it has become increasingly difficult to obtain natural clays that meet these engineering characteristics in recent years. At the same time, the cracking of compacted clay liners (CCLs) can cause a decline in their engineering properties, which is caused by uneven settlement or drying [10,11,12,13,14]. In the past decades, many natural and artificial materials have been added to clay to change the engineering characteristics and raise the performance of CCLs. AL-RAWAS et al. [15] studied the permeability of attapulgite clay–sand and found that the permeability of 70% clay–sand mixtures was lower than that of pure clay and could meet the landfill liner requirements. Nath et al. [16] carried out a range of geotechnical experiments over bentonite-modified kurna soils. The results revealed that the soil reduced its permeability and increased its unconfined compressive strength (UCS) following the addition of bentonite. Musso et al. [17] investigated the elimination of Cu^2+^ from calcareous mudstone using batch adsorption and soil column tests. The outcomes indicated that the mudstone was able to rapidly remove Cu^2+^ from the aqueous solution. The maximum adsorption capacity of Cu^2+^ by mudstone is 103.24 mg/g. Li et al. [7] investigated the viability of shale–clay mixtures as modified liner materials and found that adding shale increased the adsorptive properties of the liner material for Zn^2+^, Cd^2+^, Pb^2+^, and Cr^2+^. The maximum adsorption capacity of Zn^2+^, Cd^2+^, Pb^2+^, and Cr^2+^ for 30% shale and 70% clay is, respectively, 6.684 mg/g, 8.598 mg/g, 10.142 mg/g, and 5.371 mg/g. Aswathy et al. [6] verified that a bentonite–lithomargic clay mixture is an appropriate alternate barrier material through a saturated hydraulic conductivity performance test, UCS test, and ammonia adsorption test. With the addition of bentonite, the maximum adsorption capacity of ammonia by bentonite–lithomargic clay can reach 8220 mg/kg. Guney et al. [18] investigated the feasibility of utilizing zeolite, sepiolite, rich kaolinite, and mixtures thereof as bottom-liner materials. The investigations indicated that they were effective in improving the metal adsorption capacity, swelling potential, and strength of the clay mixture as the content of sepiolite increased. Artificial materials including fly ash, cement, lime, etc., were also utilized to enhance the engineering properties of the liner material [19,20,21]. It is worth noting that fly ash can enhance the adsorption of heavy metals; nevertheless, it may contain heavy metals and risk contaminating the environment [22]. Cement and lime are effective in improving the geotechnical properties of the liner but can cause environmental pollution when used [23].

In recent decades, nanomaterials have been extensively used in biomedical, electronic, and energy fields owing to their special chemical and physical properties. Multi-walled carbon nanotubes (MWCNTs) have very excellent mechanical properties and have been used within the field of geotechnical engineering. Loures et al. [24] found that nano clay materials prepared by the simple mixing of MWCNTs with clay could be used as flexible pavement materials through geotechnical testing. Hussain et al. [25] found that the internal friction angle, cohesion, and UCS of gypsum soil treated with MWCNTs could be increased by 78%, 232%, and 414%, respectively. Chen et al. [26] investigated the drying and shrinkage curves of MWCNT-modified bentonite and found that MWCNTs were able to inhibit the cracking of bentonite due to wet and dry cycling. Additionally, MWCNTs have a substantial specific surface area and absorptive properties for a variety of ions and organic matter [27]. Matos et al. [28] investigated the potential of MWCNTs in immobilizing the heavy metals Pb^2+^, Cu^2+^, Zn^2+^, and Ni^2+^ in soil, and found that while the addition of MWCNTs had little effect on Pb^2+^ and Cu^2+^, it could decrease the migration of Zn^2+^ and Ni^2+^. The immobilization capacity of the soil for Zn^2+^ and Ni^2+^ was increased by 30% due to the addition of MWCNTs. Li et al. [29] studied the adsorption properties of MWCNTs for Pb^2+^ at different PH. The results showed that the adsorption of MWCNTs for Pb^2+^ satisfied the single-layer adsorption and the adsorption amount increased with the increase in pH level. When the pH level was 7, the adsorption capacity of Pb^2+^ by MWCNTs reached the maximum, which was 49.95 mg/g. Iannazzo et al. [30] developed dendrimer-functionalized MWCNTs and found that they were effective for the adsorption of Hg^2+^.

A review of these studies suggests that MWCNTs can effectively improve soil engineering properties. Most of the published research has focused on the use of MWCNTs as contaminant adsorbents or centered on the impact of MWCNTs on geotechnical characteristics. There are limited studies on the utilization of MWCNT-modified clays as landfill liners and their adsorption properties for heavy metals in leachate. In this paper, we evaluate and discuss the feasibility of using MWCNT-modified clay as a bottom liner material. The engineering properties of the MWCNT-modified clay were evaluated by a range of indoor experiments, including compaction tests, variable-head permeability tests, and triaxial shear tests. The microstructure of the MWCNT-modified clay was observed using a scanning electron microscopy (SEM), and its mechanism is explained. The attenuation effect of the MWCNT-modified clay on common heavy metal ions in landfill leachate was investigated by batch adsorption tests.

## 2. Materials

### 2.1. Experimental Clay

**Figure 1:**
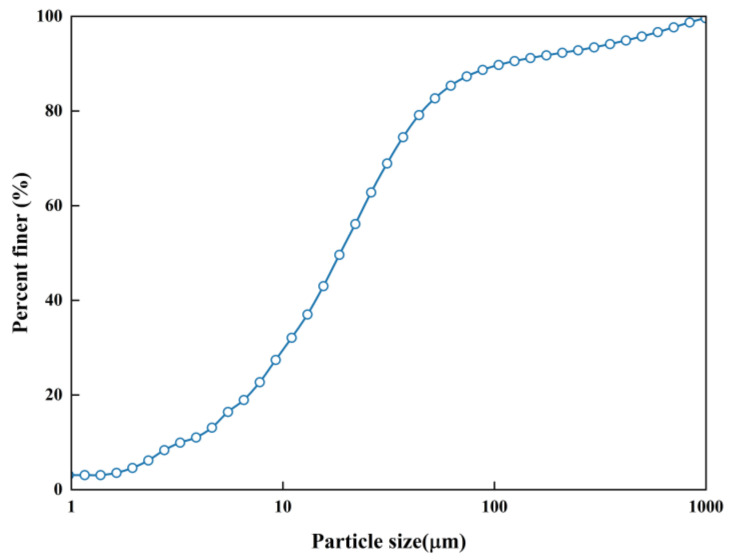
Grain size distribution of the tested clay.

The clay specimens for the trial were collected from Changzhou, China. The collected clay was carried to the geotechnical laboratory for additional tests. The clay was dried in a 105 °C oven for about 24 h and chilled to chamber temperature. It was then pulverized and sieved using a 2 mm sieve. A number of basic experiments were carried out on the basis of the Standard for Geotechnical Testing Method (GB/T 50123-2019) [31]. The results are displayed in Table 1 and Figure 1.

### 2.2. Multi-Walled Carbon Nanotubes

**Figure 2:**
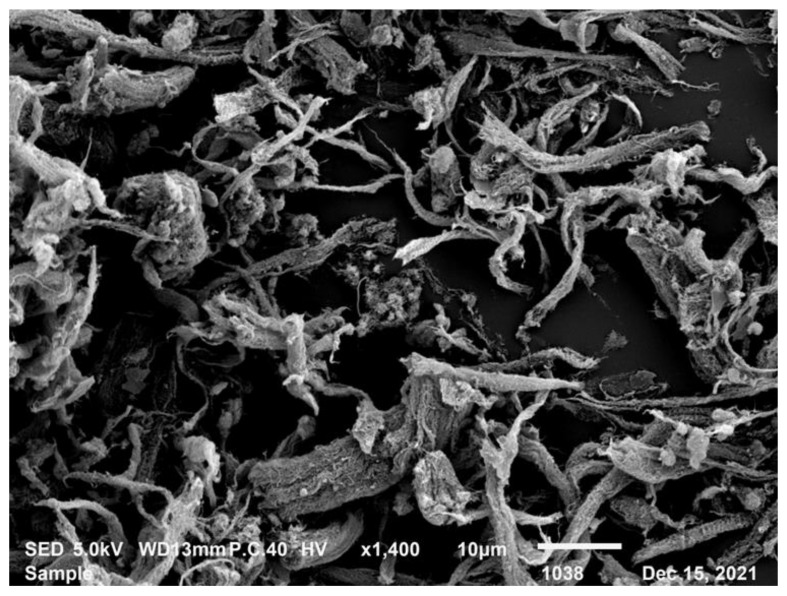
Microstructure image of MWCNTs.

The MWCNTs used in this research are GT-205 (produced by Shandong Dazhan Nanotechnology Co., Shandong, China) and Table 2 displays their physical properties. The MWCNTs are in the form of a black powder. The structure is observed using the SEM, as displayed in Figure 2. Its tube wall is spiral-like and has defects.

### 2.3. Salt Solutions

The simulation of waste leachate was generated using the dissolved heavy metals ions Cd^2+^, Cu^2+^, and Mn^2+^, which are commonly found in landfill leachate [32]. Salt solutions CdCl_2_, CuCl_2_, and MnCl_2_·4H_2_O were used to determine the adsorption capability of MWCNT-modified clay. A total of 1.631 g CdCl_2_, 2.116 g CuCl_2_, and 3.602 g MnCl2·4H_2_O were dissolved in distilled water as well as prepared as a stock solution of heavy metal ions at a concentration of 1000 mg/L. Various concentrations were acquired through dilution of a 1000 mg/L heavy metal ion solution. All compounds used in the test (purchased from Tianjin Zhiyuan Chemical Analysis Co., Ltd., Tianjin, China) were of analytical grade.

## 3. Methods

### 3.1. Compaction Tests

According to ASTM D698-12 [33], the Proctor compaction tests were performed on modified clays containing 0%, 0.5%, 1%, and 2% MWCNTs (MWCNTs mass/dry clay mass). The compaction device consists of a cylindrical mold with a diameter of 100 mm and a height of 127 mm and a drop hammer with a weight of 2.5 kg. To obtain the energy required for compaction, the drop hammer was dropped from a height of 305 mm with 25 strokes per layer for a total of three layers of compaction. The relationship between the moisture content and dry density of various specimens was obtained based on the compaction test. 

### 3.2. Variable-Head Permeability Tests

**Figure 3:**
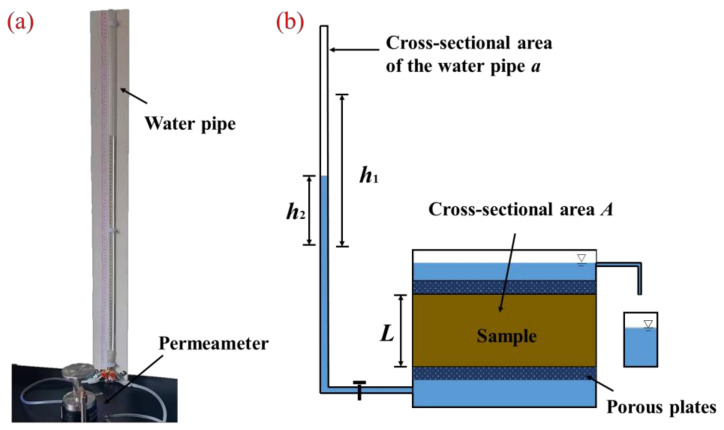
(**a**) photos and (**b**) sketches of the variable-head permeability instrument.

As per the compaction test results, cylindrical samples of 40 mm in height and 61.8 mm in diameter were made from the modified clay mixtures with different MWCNT contents at their optimum moisture content (OMC) and maximum dry density (MDD). According to ASTM D5084-16a [34], the specimens were then installed into the TST-55 permeameter (Nanjing Ningxi Soil Instrument Co., Ltd., Nanjing, China) for the variable-head permeability test (Figure 3). The tests were conducted indoors at 20 °C and the water temperature was maintained at the same temperature as the room temperature. The permeability coefficient of each specimen can be calculated by corresponding to the change in water head using Equation (1) [35]:

(1) $$k=\frac{aL}{A(t_{2}-t_{1})}\ln(\frac{h_{1}}{h_{2}})$$

where *k* is the permeability coefficient (cm/s), *a* is the water pipe’s cross-sectional area (cm^2^), *L* is the specimen’s height (cm), *A* is the specimen’s cross-sectional area (cm^2^), *t*_2_ is the time at the end of the test (s), *t*_1_ is the time at the beginning of the test (s), *h*_1_ is the initial height of the head (cm), and *h*_2_ is the final height of the head (cm).

### 3.3. Triaxial Compression Tests

**Figure 4:**
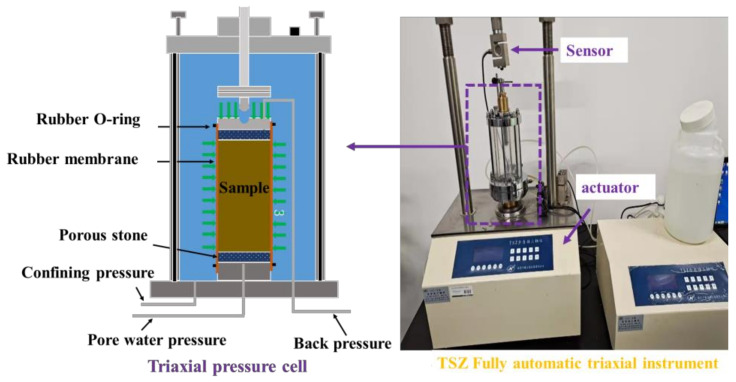
Instruments used for UU tests.

As shown in Figure 4, unconsolidated and undrained tests (UU tests) were conducted on specimens with a height of 80 mm and a diameter of 39.1 mm using the TSZ fully automated triaxial instrument (Nanjing Ningxi Soil Instrument Co., Ltd.) to assess the impact of different MWCNT contents on the mechanical characteristics of the specimens. According to ASTM D2850-23 [36], the strain rate was set to 1 mm/min, and the confining pressures were 100, 200, 300, and 400 kPa. The test was stopped when the strain reached up to 20%.

### 3.4. SEM Tests

The microstructure of the MWCNT-modified clay was observed using SEM tests to understand the reinforcement mechanism of the MWCNTs on the clay. Firstly, the specimens were cut into 5 × 5 × 10 mm soil strips and quickly frozen in liquid nitrogen. Next, the specimens were vacuum-dried in a freeze dryer for 24 h at −50 °C [37]. The freeze-dried specimens can be used for SEM tests. Subsequently, the prepared specimens were broken in the middle and placed into a vacuum ion-sputtering apparatus for gold plating with a thickness of 5 nm to improve the electrical conductivity of specimens [38]. Finally, the specimens were evaluated and an SEM experiment was conducted (Super 55 manufactured by Carl Zeiss Group, Oberkochen, Germany). 

### 3.5. Batch Adsorption Tests

According to ASTM D4646-16 [39], the adsorption performance of the clay and modified clay with different MWCNT contents was evaluated. Various concentrations of heavy metal ion solution were obtained by diluting the stock solutions at 50, 100, 200, and 400 mg/L. A total of 1 g of the sample and 100 mL of a known initial concentration of heavy metal ion solution were added to a 150 mL conical flask. The samples were then spun at 200 rpm for 24 h and centrifuged at 6000 rpm for 10 min at a constant temperature of 25°. Subsequently, the supernatant night was collected and analyzed for heavy metal concentrations using an inductively coupled plasma emission spectroscopy (ICP-OES) (Avio 550 MAX). Finally, the amount of adsorbed heavy metal ions was obtained through calculating using Equation (2) [40].

(2) $$Q_{e}=\frac{V(C_{i}-C)}{m}$$

where *Q_e_* is the equilibrium adsorption, *V* is the volume of the heavy metal ion solution, *C_i_* is the heavy metal ion concentration before adsorption, *C* is the heavy metal ion concentration after adsorption, and *m* is the mass of MWCNT-modified clay.

## 4. Results and Discussion

### 4.1. Compaction Characteristics

**Figure 5:**
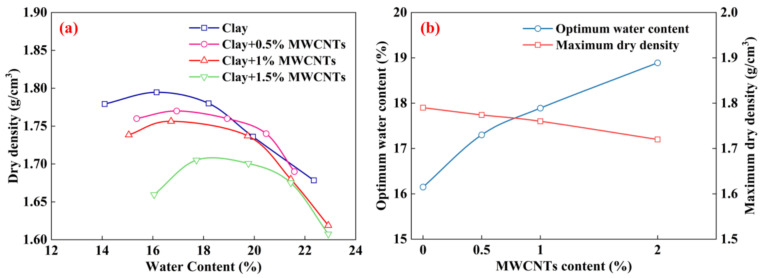
Compaction curves (**a**) and optimum compaction parameters (**b**) of clay modified with various MWCNT contents.

Figure 5 displays the results of the Proctor compaction tests on the modified clays with various MWCNT contents. For the clay specimen, its MDD is 1.79 g/cm^3^, and the corresponding OMC is 16.15%. For the MWCNT-modified specimens, the OMC of the samples increases significantly with the increase in MWCNTs. Adding 2% MWCNTs to the clay increases the OMC from 16.15% to 18.89%, which is an increase of about 17%. Although the main component of the MWCNTs was hydrophobic carbon (C), its large specific surface area provided a site for the hydration of clay particles, which facilitates the formation of hydration films; this ultimately led to an increase in the OMC. It can also be observed that the MWNCTs lead to a slight decline in the MDD. With the increase in MWCNTs, the MDD decreased from 1.79 g/cm^3^ to 1.72 g/cm^3^. This was caused by the difference in specific gravity between the clay and the MWCNTs. The specific gravity of the MWCNTs was marginally smaller than that of the clay, and the higher the content of MWNCTs, the lower the density of the MWCNT-modified clay.

### 4.2. Variation of the Permeability Coefficient

**Figure 6:**
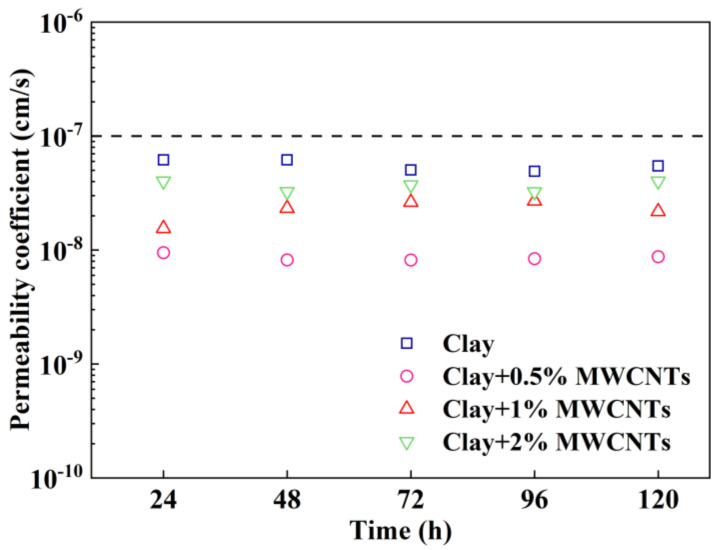
Change in permeability coefficient of modified clay with various MWCNT contents.

According to GB 50869-2013 [41], the permeability coefficient of the compacted clay liner or modified clay liner cannot be greater than 1 × 10^−7^ cm/s in the landfill. The permeability coefficients of modified clays with various MWCNT contents are displayed in Figure 6. The permeability coefficient falls rapidly and then rises slowly as the content of MWCNTs increases. For samples containing 0%, 0.5%, 1%, and 2% MWCNTs, the average permeability coefficients are 5.56 × 10^−8^ cm/s, 8.62 × 10^−9^ cm/s, 2.28 × 10^−8^ cm/s, and 3.66 × 10^−8^ cm/s, respectively. The impacts of the MWCNTs on the permeability properties of the specimens were mainly as follows. Firstly, the MWCNTs filled some of the internal pores of the clay, thereby increasing the path of water migration. Secondly, the MWCNTs were a hydrophobic material, and it was more difficult for the water migration process to pass through the interface between the MWCNTs and clay. The above two reasons explain that the MWCNT-modified clay specimens had a lower permeability coefficient than the clay specimens. When the content of MWCNTs is 0.5%, the permeability coefficient is minimized. When the MWCNT content is larger than 0.5%, the permeability coefficient increases with the increase in MWNCT content. This was mainly due to the fact that MWCNTs are nano-sized materials, which would inevitably agglomerate as the content increases. The agglomeration of MWCNTs led to the inability of clay particles to contact each other, increasing the internal pores of the sample, and ultimately increasing the permeability coefficient. Although the continued addition of MWCNTs caused the permeability coefficients to increase, the samples with 1% and 2% MWCNTs still had permeability coefficients that were less than those of the clay specimens and also met the permeability requirements of the modified clay liner.

### 4.3. Variation in Mechanical Properties

**Figure 7:**
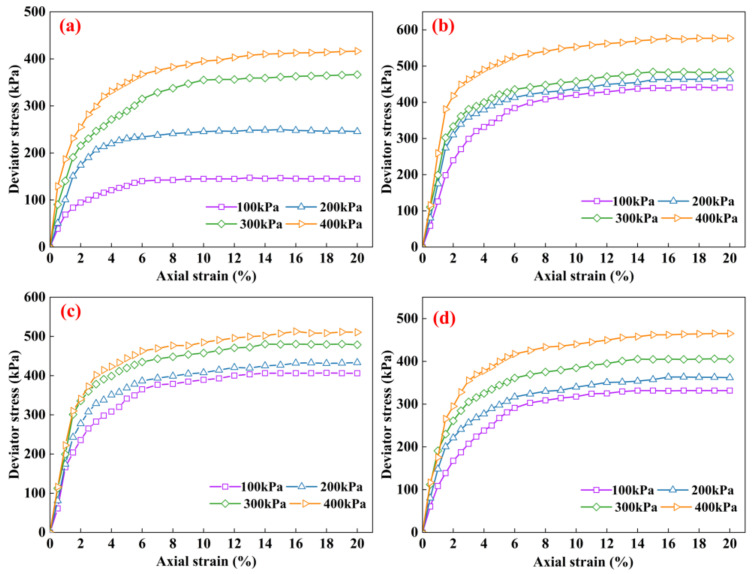
Stress–strain curves of clay modified with different MWCNTs contents: (**a**) 0%, (**b**) 0.5%, (**c**) 1%, (**d**) 2%.

Figure 7 demonstrates the stress–strain curves of the modified clays with various MWCNT contents. The axial strain is in the range of 0% to 4.0%, and the deviation stress curve rises sharply. As the axial strain rises (>4.0%), the curve slowly increases. The confining pressure has a significant impact upon the stress–strain curve of the samples. For the clay samples, the stress–strain curves show weak strain softening at confining pressures of 100 kPa and 200 kPa. At confining pressures of 300 kPa and 400 kPa, the stress–strain curves show strain hardening. A high confining pressure enhanced the interlocking forces between the clay particles, increased the positive stresses on the relative slip surfaces (i.e., shear surfaces), and enhanced the deformation resistance of the clay. For the MWCNT-modified clay samples, the stress–strain curves all show strain hardening. This was due to the fact that the MWCNTs that were randomly distributed in the clay constituted a three-dimensional network structure, which not only strengthened the potential weak surfaces in the clay but also reduced the cracks in the clay and improved the plasticity of the clay samples.

**Figure 8:**
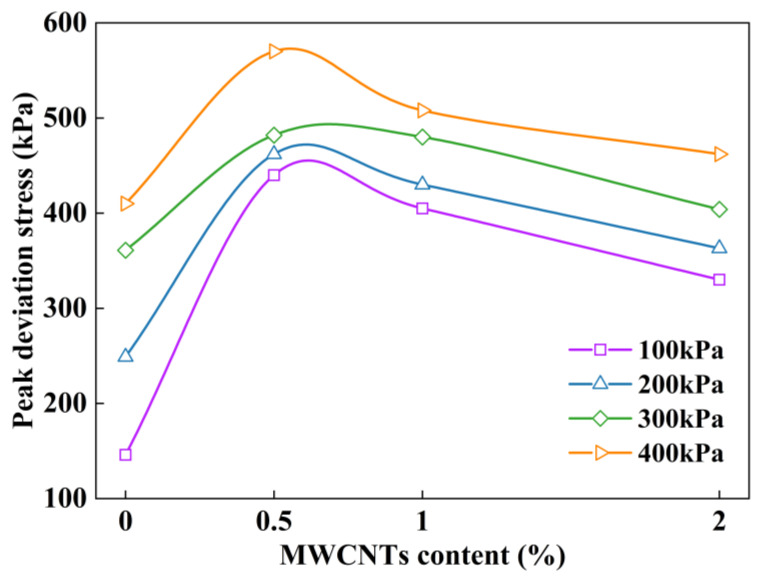
Peak deviation stresses in clay modified with different MWCNT contents.

The peak deviation stress of the specimens is a critical index to characterize their load-carrying ability. Considering the difference between stress–strain curves, for strain-softening curves, the maximum point is the peak deviation stress. For strain-hardening curves, the deviation stress at a 20% strain is taken as the peak deviation stress [42]. Figure 8 shows the peak deviation stress of the MWCNT-modified clay specimens under various confining pressures. With the increase in MWCNTs, the peak deviation stress shows a tendency for a large increase and then a slow decrease. This was mainly attributed to the fact that the MWCNTs filled the internal voids of the clay and improved the clay’s compactness, while its own adsorption of clay enhanced the cementation effect, and the peak deviation stress became larger. With the increased content of MWCNTs, the lubricating effect of the MWCNTs was stronger than the cementing effect, resulting in the reduction of the peak deviation stress.

**Figure 9:**
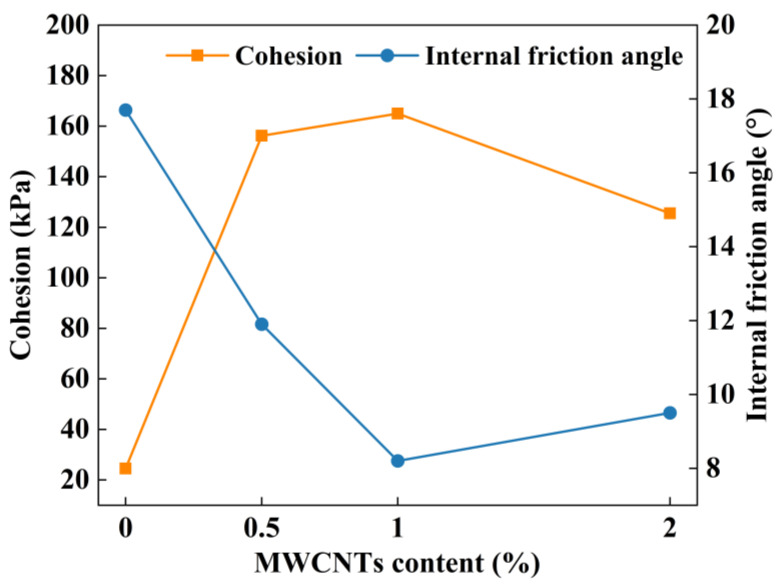
Cohesion and angle of internal friction of modified clays with different contents of MWCNTs.

In order to additionally examine the impact of the MWCNTs on the shear strength parameters of clay specimens, the internal friction angle and cohesion of various MWCNT-modified clays were calculated using the Mohr–Coulomb theory [43], as shown in Figure 9. The cohesion of the specimens modified with different MWCNT contents are 24.5 kPa, 156.2 kPa, 165 kPa, and 125.5 kPa, respectively. The highest point of the curves appears when the content of MWCNTs is in the range of 0~1%, and the cohesion reaches the maximum value. The curves show a slow decreasing tendency, and the cohesion decreases when the content of MWCNTs is in the range of 1~2%, but it is still significantly larger than that of the clay specimens. When the content of MWCNTs did not reach 1%, the inhibition effect of the frictional resistance and occlusal force at the interface between the MWCNTs and clay particles on the occurrence of misalignment and shear displacement of the clay particles increased as the MWCNTs increased. When the content of MWCNTs was greater than 1%, the MWCNTs were more easily agglomerated, and the distribution uniformity in the clay was progressively reduced, thus degrading the effective contact effect between the MWCNTs and clay particles and hindering the agglomeration of the clay particles, leading to a rise in the porosity of the clay, and reducing the cohesion of the specimens to a certain extent. The internal friction angles of the specimens modified with different MWCNT contents are 17.7°, 11.9°, 8.2° and 9.5°, respectively. When the content of MWCNTs is between 0% and 1%, the angle of internal friction falls with the increase in MWCNTs. When the content of MWCNTs is 1~2%, the change in the internal friction angle was not large, which was attributed to the fact that the OMC of the specimen increased with the increase in MWCNTs, and the bonded water film in the specimen became thicker and played a lubricating role; thus, the internal friction angle was reduced. On the contrary, the increase in MWCNTs also increased the point-to-point contact between the MWCNTs and clay particles, and the friction increased. When the content of MWCNTs was less than 1%, the lubrication played a dominant role, and the internal friction angle gradually decreased. When the content of MWCNTs was greater than 1%, the point-to-point contact force played a dominant role and the angle of internal friction increased slightly.

**Figure 10:**
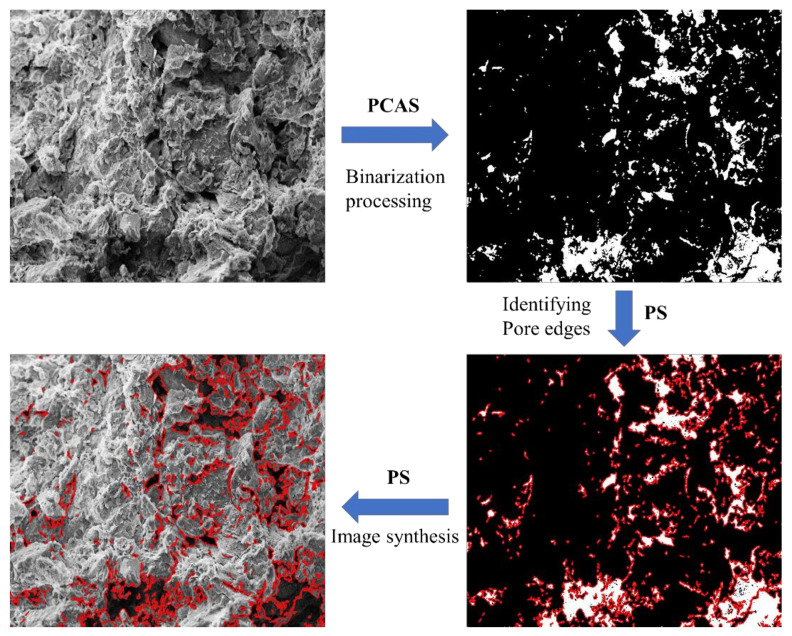
SEM image-processing procedure.

The SEM images were processed using the Pore and Crack Analysis System (PCAS) [44]. The images were first binarized after selecting a suitable threshold [45]. The black areas in the binarized image represented particles and the white areas represented pores. Then the edges of the pores were identified and marked with red lines using Adobe Photoshop CC 2018 (PS). Finally, the red marked line in the binarized image was synthesized with the SEM image and the process is displayed in Figure 10.

**Figure 11:**
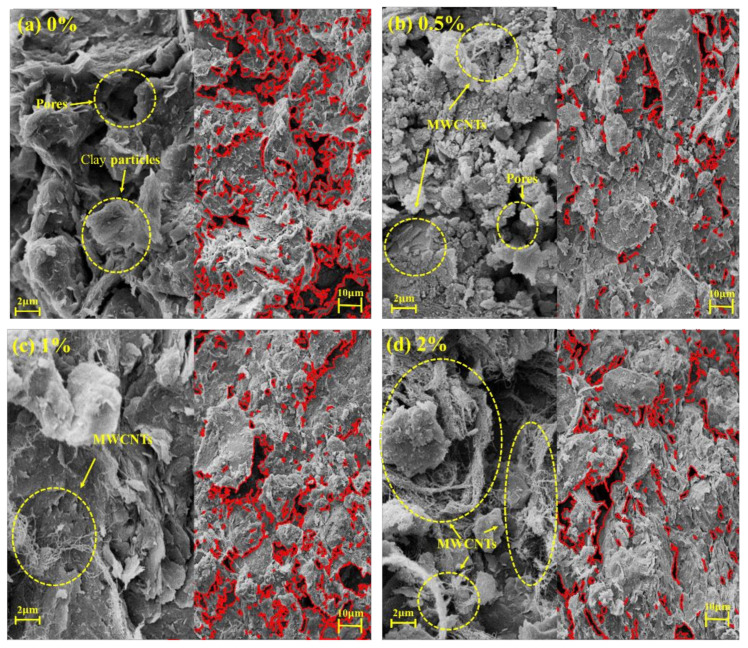
SEM images of samples with different MWCNT contents: (**a**) 0%, (**b**) 0.5%, (**c**) 1%, and (**d**) 2%.

Figure 11 illustrates images of the specimen with various MWCNT contents magnified 10,000 and 2000 times. Firstly, the sample with 0% MWCNTs is observed (Figure 11a), and the clay particles are flaky. Particle-to-particle contact is dominated by face-to-face contact, as well as a small amount of point-to-face contact. Pores are present between the point–face contacts. With the addition of MWCNTs (Figure 11b), clay particles form aggregates with MWCNTs as the core, forming a denser structure. Cracks in the soil are reduced, and micropores are increased. A very small amount of MWCNTs can also be observed to aggregate. At this time, soil–particle contact is still mainly face-to-face contact, with a small amount of point-to-face contact. When the content of MWCNTs is 1% (Figure 11c), it can already be observed that the MWCNT agglomeration causes a decrease in the contact between the particles, and the large pores start to increase. This is because the three-dimensional network structure formed by the MWCNTs can strengthen the soil structure, but excessive aggregation leads to a reduction in the indirect contact force of soil particles, forming weak faces inside the soil. When the content of WMCNTs is 2% (Figure 11d), the contact between the clay particles is hindered by excessive MWCNT agglomeration, and the number of macropores further increases.

**Figure 12:**
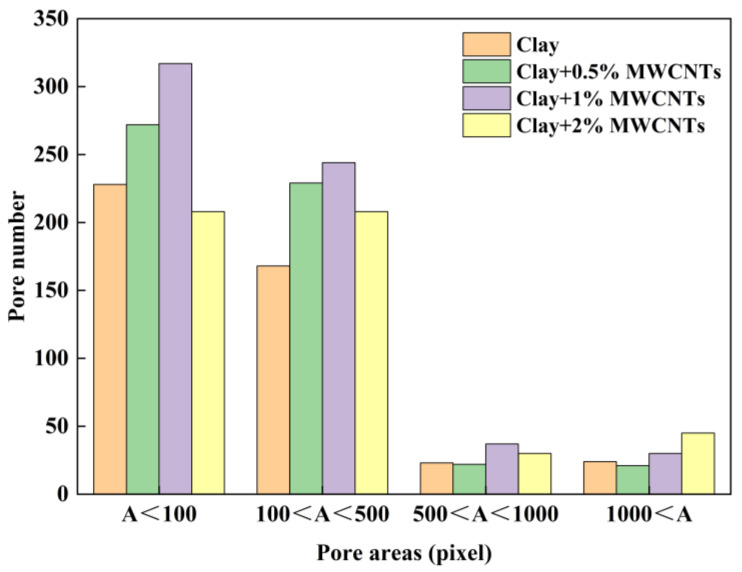
PCAS processing SEM image results.

In order to further quantitatively characterize the effect of the MWCNTs on the number of pores in the specimens, the PCAS was utilized to identify the pores in the images and the pore areas were counted (Figure 12). Pore sizes are categorized by area as micropores, small pores, medium pores, and large pores, and their corresponding areas A corresponded to A < 100 pixels, 100 pixels < A < 500 pixels, 500 pixels < A < 1000 pixels, and 1000 pixels < A. With the increase in MWCNT content, the number of micropores in the sample was 228,272,317 and 208, respectively. The number of small pores was 168,229,244 and 208, respectively. The number of micropores and small pores increased first and then decreased with the increase in MWCNT content. When the content of MWCNTs was 1%, the number of micropores and small holes reached the maximum. The changes in mesopores and macropores are significantly different from those of micropores and micropores. When the content of MWCNTs was between 0% and 1%, the number of medium and large pores in the sample first decreased and then increased. When the content of MWCNTs was between 1% and 2%, the middle pores in the sample began to decline, while the large pores continued to increase. The proper amount of MWNCTs can effectively fill the internal pores of the soil, thereby reducing the cracks inside the sample and strengthening the soil. This also explains the increase in the shear strength and the decrease in the permeability coefficient with the addition of MWCNTs. Then, an excess of MWCNTs led to the weakening of the contact force of the clay particles, which weakened the soil structure and increased the number of medium and large pores in the soil. Finally, the shear strength of the sample decreased slightly, and the permeability coefficient increased.

### 4.4. Heavy Metal Ion Adsorption Results

The adsorption capacity (*Q*_e_) and equilibrium concentration (*C*_e_) of the adsorbent for different heavy metal ions at equilibrium were determined by batch adsorption tests. The Freundlich isotherm and Langmuir isotherm were applied to evaluate the extent of the adsorption of heavy metal ions in the solution by the adsorbent at equilibrium.

The Freundlich isotherm, which is predicated on multilayer adsorption and predicts inhomogeneous adsorption, is defined as follows [46]:

(3) $$\ln Q_{e}=\ln K_{F}+\frac{1}{n}\ln C_{e}$$

where *K_F_* is the adsorption constant (L/g) and n is the adsorption strength. Generally, 0 < 1/n < 1 represents a favorable adsorption.

The Langmuir isotherm, which is based on monolayer adsorption with a uniform distribution of adsorbent active sites, has the following definition [47]:

(4) $$\frac{C_{e}}{Q_{e}}=\frac{1}{K_{L}\cdot Q_{\max}}+\frac{1}{Q_{\max}}C_{e}$$

where *K_L_* is the isotherm parameter (L/g) and *Q_max_* is the adsorption maximum (mg/g).

The Freundlich isotherm and Langmuir isotherm used in this study are often used to assess the adsorption capability of clay. The outcomes of the adsorption tests of the MWCNT-modified clay on different heavy metal ions are shown in Table 3.

**Figure 13:**
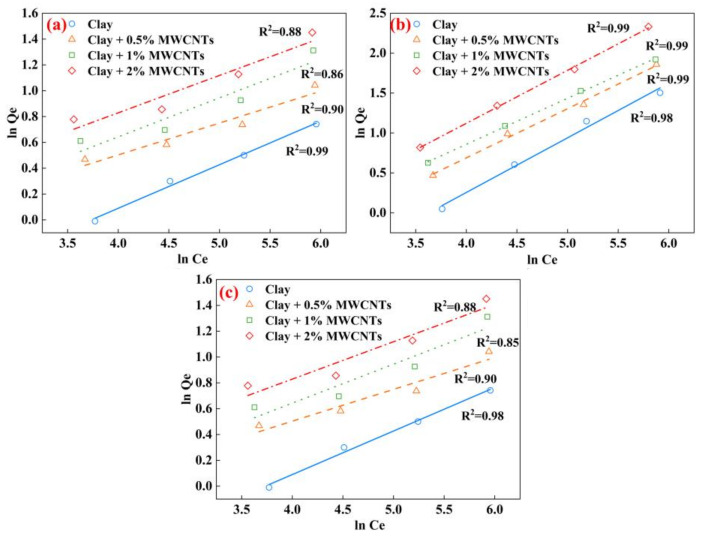
Freundlich adsorption isotherms of different heavy metal ions (**a**) Cu^2+^, (**b**) Cd^2+^, (**c**) Mn^2+^.

Figure 13 shows the experimental results of fitting Cu^2+^, Cd^2+^, and Mn^2+^ separately using the Freundlich isotherm. In order to evaluate the adsorption parameters, the adsorption isotherms are shown in a linearized form. For Cu^2+^, the *K_F_* and *n* of the clay are 3.52 and 2.97. The *K_F_* and *n* of the clay modified with 0.5% MWNCTs are 1.62 and 4.07. The *K_F_* and *n* of the clay modified with 1% MWNCTs are 1.76 and 3.31. The *K_F_* and *n* of the clay modified with 2% MWNCTs are 1.39 and 3.46. The increase in the *n* value with the content of MWCNTs reveals the favorable adsorption of Cu^2+^ by MWCNTs. From Figure 12b,c, MWCNTs also favor the adsorption of Cd^2+^ and Mn^2+^.

**Figure 14:**
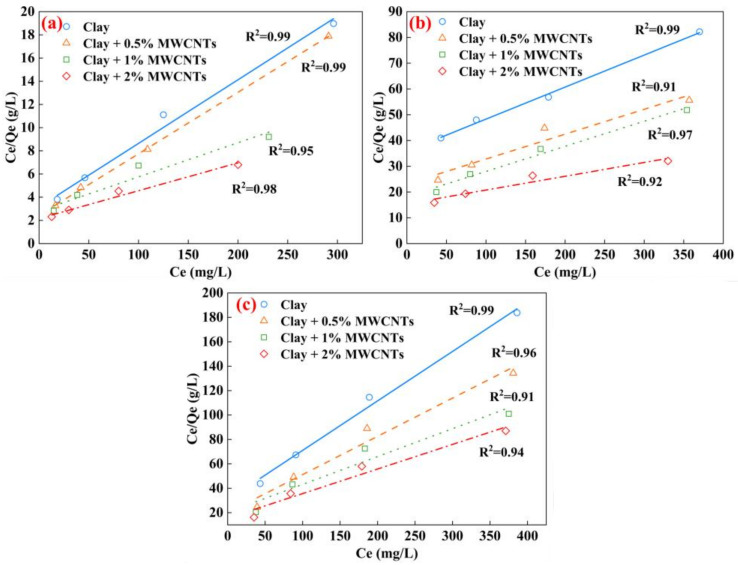
Langmuir adsorption isotherms of different heavy metal ions (**a**) Cu^2+^, (**b**) Cd^2+^, (**c**) Mn^2+^.

Compared to the Freundlich isotherm, the Langmuir isotherm provides a better fit to the experimental results for Cu^2+^, Cd^2+^, and Mn^2+^ (Figure 14), which also demonstrates that the clay tends to adsorb heavy metal ions in a monolayer. Similarly, the adsorption isotherms are shown in a linearized form. For Cu^2+^, the maximum adsorption (*Q_max_*) of the samples with different MWCNT contents are 18.22 mg/g, 18.86 mg/g, 33.93 mg/g, and 41.67 mg/g, respectively. The addition of 2% MWNCNTs caused a 228% enhancement in the adsorption of Cu^2+^ by the samples. For Cd^2+^, the *Q_max_* scores are 8.05 mg/g, 10.36 mg/g, 10.26 mg/g and 18.69 mg/g. The addition of 2% MWNCNTs enhanced the adsorption of Cd^2+^ by 124%. For Mn^2+^, the *Q_max_* scores are 2.46 mg/g, 3.19 mg/g, 4.38 mg/g and 4.97 mg/g. The addition of 2% MWNCNTs enhanced the adsorption of Cd^2+^ by 202%. The preference of the clay for heavy metal ion adsorption was Cu^2+^ > Cd^2+^ > Mn^2+^. The clay surface adsorption of heavy metal ions was limited; when its surface-active sites were completely taken up, no additional adsorption would occur. The MWCNTs’ large specific surface area enabled the MWCNT-modified clay to greatly enhance the adsorption of heavy metal ions.

### 4.5. Comparison with Other Liner Materials

To evaluate the effectiveness of the MWCNT-modified clay as a landfill liner material, 0.5%MWCNT-modified clay was compared with other materials, and the results are shown in Table 4. The ultimate strength, permeability coefficient, and maximum adsorption capacity of heavy metal ions were compared. The MWNCT-modified clay had excellent mechanical properties, and its ultimate strength was better than that of bentonite–lithomargic clay. The reason for this is that MWCNTs not only act as a core to make the aggregates between the clay particles denser, but also, the three-dimensional network structure formed by the MWCNTs strengthens the soil structure, which makes the soil withstand greater external forces without damage. Although the permeability coefficient of the MWCNT-modified clay is slightly larger than that of bentonite–lithomargic clay, it is still much smaller than that of clay shale. In the adsorption of heavy metal ions, the performance of the MWCNT-modified clay is particularly excellent. The maximum adsorption capacity of Cr^2+^ by the MWCNT-modified clay is 2.46 times greater than that of clay shale. In conclusion, the MWCNT-modified clay has strong potential as a landfill liner material.

## 5. Conclusions

Landfill bottom liner materials typically use a compacted clay liner to limit the leachate contamination of the underlying soil and groundwater. However, natural clay that meets the liner material requirements is becoming increasingly difficult to obtain. The feasibility of MWCNT-modified clay as a liner material was investigated using a comprehensive experimental method. On the basis of the outcomes of the research, the following conclusions can be drawn:

(1) With the increase in MWCNT content, the geotechnical properties of the MWCNT-modified clay have been improved. Its permeability coefficient decreases and the peak deviation stress increases.
(2) From the SEM images, the variation in the number of pores with different areas inside the clay specimens with MWCNT content was the main reason for the variation in their permeability and shear strength.
(3) The preference of clay for the adsorption of heavy metal ions was Cu^2+^ > Cd^2+^ > Mn^2+^. The maximum adsorption of Cu^2+^, Cd^2+^, and Mn^2+^ by the modified clay was enhanced by 228%, 124%, and 202% when the MWCNT content was 2%, respectively.

## Figures and Tables

**Table 1:** Physical properties of clay.

Clay Properties	Description
Plastic Limit (%)	16.1
Liquid Limit (%)	40.2
Plasticity Index (%)	24.1
Optimum Moisture Content (%)	16.1
Maximum Dry Density (g/cm^3^)	1.79
Natural Moisture Content (%)	21.2
Specific gravity	2.73

**Table 2:** Physical properties of MWCNTs.

Length (nm)	Diameter (μm)	Ash Content (%)	Purity (%)	Specific Surface Area (m^2^/g)	Apparent Density (g/cm^3^)
5–50	10–20	<2	>85	160–210	0.06–0.1

**Table 3:** Adsorption parameters of Freundlich isotherm and Langmuir isotherm.

Type	Heavy Metal Ion	Freundlich Isotherm			Langmuir Isotherm		
		*K*_F_ (L/g)	*n*	R^2^	*K*_L_ (L/g)	*Q_max_* (mg/g)	R^2^
Clay	Cu^2+^	3.52	2.97	0.99	0.017	18.22	0.99
	Cd^2+^	11.97	1.46	0.98	0.003	8.05	0.99
	Mn^2+^	3.52	2.97	0.99	0.013	2.46	0.99
Clay + 0.5%MWCNTs	Cu^2+^	1.62	4.07	0.90	0.021	18.86	0.99
	Cd^2+^	5.90	1.62	0.99	0.004	10.36	0.91
	Mn^2+^	1.62	4.06	0.90	0.015	3.19	0.96
Clay + 1%MWCNTs	Cu^2+^	1.76	3.31	0.86	0.010	33.93	0.95
	Cd^2+^	4.27	1.73	0.99	0.005	10.26	0.97
	Mn^2+^	1.62	3.22	0.85	0.011	4.38	0.91
Clay + 2%MWCNTs	Cu^2+^	1.39	3.46	0.88	0.011	41.67	0.98
	Cd^2+^	4.63	1.51	0.99	0.003	18.69	0.92
	Mn^2+^	1.39	3.46	0.88	0.013	4.97	0.94

**Table 4:** Comparison with other liner materials.

Type	Ultimate Strength (kPa)	Permeability Coefficient (cm/s)	Heavy Metal Ions	*Q_max_* (mg/g)
Clay + MWCNTs	570	8.62 × 10^−9^	Cu^2+^	41.67
			Cd^2+^	18.69
			Mn^2+^	4.97
Bentonite + lithomargic clay [6]	420	6.83 × 10^−11^	-	-
Clay + Shale [7]	-	7.94 × 10^−7^	Cd^2+^	8.598
			Cr^2+^	7.599
			Zn^2+^	6.684
			Pb^2+^	10.142

## Data Availability

Data are contained within the article.
